# Experiences and Expectations of Information and Communication Technologies in Flexible Assertive Community Treatment Teams: Qualitative Study

**DOI:** 10.2196/42796

**Published:** 2023-02-09

**Authors:** Erlend Bønes, Conceição Granja, Terje Solvoll

**Affiliations:** 1 Norwegian Centre for e-Health Research University Hospital of North Norway Tromsø Norway; 2 Faculty of Nursing and Health Sciences Nord University Bodø Norway

**Keywords:** mental health, FACT, electronic health records, eHealth, EHR, electronic whiteboards, community, treatment, qualitative, COVID-19, patient care, mental illness, information technology, thematic analysis, data access, information and communication solutions, ICT, Norway, semistructured interviews

## Abstract

**Background:**

Flexible Assertive Community Treatment (FACT) is a model of integrated care for patients with long-term serious mental illness. FACT teams deliver services using assertive outreach to treat patients who can be hard to reach by the health care service, and focus on both the patient’s health and their social situation. However, in Norway, FACT team members have challenges with their information and communication (ICT) solutions.

**Objective:**

The aim of this study was to explore Norwegian FACT teams’ experiences and expectations of their ICT solutions, including electronic health records, electronic whiteboards, and calendars.

**Methods:**

We gathered data in two phases. In the first phase, we conducted semistructured interviews with team leaders and team coordinators, and made observations in FACT teams targeting adults. In the second phase, we conducted semistructured group interviews in FACT teams targeting youth. We performed a thematic analysis of the data in a theoretical manner to address the specific objectives of the study.

**Results:**

A total of 8 teams were included, with 5 targeting adults and 3 targeting youth. Due to the COVID-19 pandemic, we were not able to perform observations in 2 of the teams targeting adults. Team leaders and coordinators in all 5 teams targeting adults were interviewed, with a total of 7 team members participating in the teams targeting youth. We found various challenges with communication, documentation, and organization for FACT teams. The COVID-19 pandemic was challenging for the teams and changed the way they used ICT solutions. There were issues with some technical solutions used in the teams, including electronic health records, electronic whiteboards, and calendars. Lack of integration and access to data were some of the main issues identified.

**Conclusions:**

Despite the FACT model being successfully implemented in Norway, there are several issues regarding the ICT solutions they use, mainly related to access to data and integration. Further research is required to detail how improved ICT solutions should be designed. While FACT teams targeting adults and youth differ in some ways, their needs for ICT solutions are largely similar.

## Introduction

Flexible Assertive Community Treatment (FACT) is a model of comprehensive and integrated care for patients with long-term serious mental illness [1]. FACT teams deliver services using assertive outreach to treat patients who can be hard to reach by the health care service, and focus on both the patient’s health and their social situation. The teams should consist of a psychiatrist, a psychologist, case managers, an employment specialist, an addiction specialist, and a peer support worker. In addition, the teams have a team leader and team coordinator [2]. The FACT model is a variant of the Assertive Community Treatment (ACT) model, which was developed in the United States in the 1970s [3]. FACT was developed in the Netherlands in the early 2000s and is more suited for rural areas with lower patient populations compared with ACT [3]. FACT has been highlighted as a good practice of integrated community-based mental health care, but with less conclusive evidence than available for the ACT model [4]. Some FACT teams target youth aged 12-25 years and were established to overcome the problems of traditional services, including reaching youth with complex issues, unclear responsibilities, and lack of service integration [5].

In Norway, there are approximately 70 FACT teams targeting adults [6] and 5 teams targeting youth. Most FACT teams are organized as a cooperation between specialist care and primary care within one or more municipalities. FACT teams in rural areas often include multiple municipalities and are distributed over several locations [7]. The Norwegian government has the responsibility for the specialist mental health care delivered by the hospitals or community mental health centers, owned by four Regional Health Authorities, while primary care and local services are delivered by the 356 municipalities. In 2020, Landheim et al [8] published a report evaluating Norwegian FACT teams, which showed improvement in housing and social functioning for the patients treated by these teams. The evaluation also showed that there was improvement in symptoms in many areas, but not for anxiety and depression. There was a reduction in compulsory inpatient days of 42% [8].

Some of the most relevant information and communication technology (ICT) solutions for FACT teams are the electronic health record (EHR) and electronic whiteboards. In specialist health care in Norway, most health regions use the EHR named DIPS, except for the Trøndelag region of Norway, which is in the process of implementing the Epic EHR system. However, there are several different EHR systems in use in primary care. Since most FACT teams are organized as a cooperation between specialist care and one or more municipalities, this leads to several different EHR systems being available for most teams [9]. FACT teams have daily meetings, where they go through their list of patients who require intensive follow-up to keep all team members updated on the patient’s status and further treatment plans. During these meetings, the teams use electronic whiteboards that display information about the patients [1]. Video conferencing is also used in many FACT teams, which can be used for meetings within the teams and with other partners or for video consultations with patients. In this paper, we use the term “video consultation” to refer to video conference meetings between one or more health care workers and a patient with the intent of treating the patient.

Two studies by Trane et al [7,10] examined the implementation of the FACT model in Norway. One of the studies looked at barriers for integrated care in a fragmented service system such as the Norwegian system, which showed that different digital systems, including EHR systems, represented a major barrier to integrated care [10]. This was described as a factor that could lead to errors in medication. The second study reported on the challenges when adapting the FACT model in rural areas and the adaptations to the model, highlighting that teams used video conference during team meetings and some teams used video consultations with the patients [7]. The use of video conferencing was an adaptation to make the FACT model more suited for rural areas.

The COVID-19 pandemic has affected the organization of many health care services, including FACT teams. During this pandemic, the use of telemedicine in health care increased [11]. Guan et al [12] described how a team informed by the FACT model used adaptations and innovations to minimize service disruption for their patients. This included the use of video conference during team meetings, and a shift to use of virtual visits by telephone and online telehealth platforms. One challenge was that some patients did not have access to a phone or the internet. This was mitigated by donations of phones and tablets by community resources. Couser et al [13] used the same general framework as adopted by Guan et al [12] for reporting on adaptations to the COVID-19 pandemic in an ACT team, while also discussing the findings in relation to the updated literature. They also reported a switch to telephone visits, and a minority of patients being willing and able to use Microsoft Teams for video consultations. The ACT team also used video conferencing for team meetings. The COVID-19 pandemic also affected other multidisciplinary teams in health care. Patient-centered teams treat patients in the transitional phase between hospital and primary care [14]. A study on the use of video conferencing in these teams showed that a video conference was suited for some of these patients, but there were significant challenges for other patients [15].

In 2019, standardized patient pathways were introduced for all mental health services in Norway [16]. This was done to reduce variance in treatment, ensure user participation, and improve coordination between various health services. As a result, all patients in Norwegian FACT teams should participate in a standardized patient pathway.

While the implementation of FACT teams in Norway has largely been a success, several challenges with the ICT tools used by FACT teams have been reported [9]. The aim of this study was to explore the experiences and expectations of FACT teams with the ICT tools that are relevant in their work.

## Methods

### Data Collection and Design

Computer-supported cooperative work (CSCW) [17] is a field of research examining how collaboration and coordination can be supported by computer systems. Early studies in CSCW were focused on a single workplace, which subsequently expanded to the study of multisite workplaces and mobility [18]. Ethnography has its roots in the social sciences and has been widely used within the CSCW field [19]. Some key principles in ethnography include studying a phenomenon in its natural setting, a holistic approach, providing a descriptive understanding of the phenomenon, and taking the community members’ perspectives into consideration [18]. These principles were considered to be well-suited to the goals of our data collection. Therefore, we used an ethnographic approach to data collection in the teams while working within a CSCW framework with focus on a workplace.

The data were collected in two phases. The first phase of data collection focused on FACT teams targeting adults, which included five teams. These teams were chosen based on purposeful selection [20] to collect relevant information for our research goals. We selected two urban and three rural teams in different geographical areas of Norway. We had planned to perform observations and semistructured interviews with these teams, but because of restrictions related to the COVID-19 pandemic, we were only able to complete the data collection for three teams. For the two remaining teams, we conducted interviews using Skype for Business. The data collection in the FACT teams targeting adults was completed from August 2020 to January 2021 by one researcher. We contacted the team leaders before the observations; the team leaders acted as gatekeepers, giving the researcher access to the teams. During the observations, the researcher participated in various types of team meetings. He also had informal discussions with team members and observed their use of ICT solutions. The researcher participated in three meetings with patients after obtaining their consent. These patients were chosen by the FACT teams themselves; several patients were asked to participate but chose not to meet with the researcher. During the observations, the researcher wrote memos containing thoughts, observations, and ideas. During the semistructured interviews, the team leaders and team coordinators in all five teams were interviewed one-to-one using an interview guide. The questions in the interview guide, provided in Textbox 1, were based on previous knowledge of the use of ICT tools in Norwegian FACT teams [9]. The interviews varied in length from 30 to 60 minutes. After the interview with the first team, the topic of team calendars was identified and added to the interview guide. The researcher took notes during the interviews, and clarified and expanded on them immediately after the interviews.

The second phase of the data collection focused on FACT youth teams, and the three FACT youth teams operating in Norway at the time were included. During the fall of 2021, one researcher conducted semistructured interviews with the teams with the following two goals: (1) to form the basis of a requirement specification for an electronic whiteboard for FACT youth teams in Norway, and (2) to provide a wider view of the ICT needs of FACT youth teams. All team members were invited to participate in the interviews, but not all members were able to participate. The number of participants ranged from 1 to 3, for a total of 7 participants. During the interviews, the researcher presented predefined use cases (see Textbox 2) tied to the use of the electronic whiteboard. This was used as a starting point for discussions on expectations concerning an ideal electronic whiteboard, supported by an interview guide for additional questions (see Textbox 3). The use cases and interview guide were designed based on our previous research on FACT teams targeting adults. During the interviews, the researcher took notes and wrote a memo after each interview with additional thoughts and impressions from the interviews. The interviews were recorded and transcribed.

Preliminary data analyses of the research have been published for FACT teams targeting adults [21] and youth [22]. In this paper, we present an in-depth analysis of the complete data set collected in the two phases of the study. We performed a thematic analysis [23,24] of the data following a theoretical “top-down” approach. During the observations, we gathered data on many aspects of the FACT teams; however, when analyzing the data, we focused on the specific goals of this study: the FACT teams’ experiences with and expectations of ICT solutions. This allowed for stronger focus on the aims of the study, but came at the expense of an explorative analysis of all the data. We chose this approach because it allowed us to meet the goals of the study in a better way. During the data analysis, we familiarized ourselves with the data by repeatedly reading the data set. We then coded the data relevant to the aims of our study using the software NVivo. Since the analysis was performed in a deductive manner with focus on our specific aims, only text that was relevant to our focus was coded. We defined initial themes, which were divided by codes using NVivo. Reviewing the themes, we found that it was useful to add subthemes to differentiate distinct aspects of the data. The new subthemes were added, as we found other interesting aspects of the data, or merged if they did not have sufficient codes to justify being classified as their own subthemes.

**Interview guide questions for Flexible Assertive Community Treatment (FACT) teams targeting adults.**

- What is your role in the team?
- How does access to the electronic health record work for the different team members?
- How does the team use electronic whiteboards?
- How does the team communicate internally?
- How does the team communicate with patients?
- How does the team communicate with cooperating partners?
- How are calendars used in the teams?
- What are the biggest challenges related to the use of technology?
- How can the use of technology make your work easier?
- How has the COVID-19 pandemic affected your work?

**Use cases discussed in Flexible Assertive Community Treatment (FACT) youth teams.**

- Referral of patients to FACT youth teams
- Use of the whiteboard during daily meetings
- Updating of the whiteboard after meeting a patient
- Transfer of a patient from intensive follow-up to case management
- End of treatment of a patient from the FACT youth team
- Use of the team calendars

**Interview guide questions for Flexible Assertive Community Treatment (FACT) youth teams.**

- How do you currently use the electronic whiteboard?
- Do you want to use the electronic whiteboard for other purposes?
- Is there any kind of integration between the electronic whiteboard and electronic health record solutions? If not, is this something you want?
- What team members should have access to the electronic whiteboard?
- Do you have a need to extract reports and statistics from the electronic whiteboard? If so, what kind of reports and statistics?
- What calendar solutions do the teams use? Is there a need for better calendar solutions?

### Ethical Considerations

This project has been approved by the Data Protection Official at Innlandet Hospital Trust (137877). The project was also reviewed by the Regional Ethical Committee, who deemed the project to be outside of their mandate (REK Sør-Øst 104537).

All interviewees signed informed consent forms. The data were stored securely and deidentified. No compensation was given to the participants.

## Results

### General Characteristics of Teams

Table 1 shows the characteristics of the teams and data collection methods used.

**Table 1:** Characteristics of the teams.

Team	Target group	Setting	Coverage area	Team employment	Methods used
Team 1	Adults	Rural	4 municipalities	Specialist and secondary care	Observations and interviews
Team 2	Adults	Urban	1 city district	Specialist and secondary care	Observations and interviews
Team 3	Adults	Urban	1 municipality	Specialist and secondary care	Observations and interviews
Team 4	Adults	Rural	2 municipalities	Specialist care only	Video interviews
Team 5	Adults	Rural	2 municipalities	Specialist and secondary care	Video interviews
Team 6	Youth	Urban	1 municipality	Mainly primary care	Focus group interview
Team 7	Youth	Urban	1 city district	Specialist and secondary care	Focus group interview
Team 8	Youth	Urban	1 city district	Specialist and secondary care	Focus group interview

### Themes

#### Overview

Five main themes were identified with eight subthemes. The main themes were “Communication,” “Documentation,” “Organization,” “COVID-19,” and “Technologies.” The main themes and subthemes are presented in Table 2 and described in turn below.

**Table 2:** Themes of data.

Main themes	Subthemes
Communication	Suitability of video consultations
Documentation	Documentation while traveling
Organization	Systems not adapted to FACT^a^ teams
COVID-19	Maintaining team capacity, maintaining contact with patients
Technologies	Electronic health records, electronic whiteboards, calendar, lack of integration

^a^FACT: Flexible Assertive Community Treatment.

#### Communication

The main theme Communication includes codes related to the communication needs of the FACT teams. This includes communication with patients, partners, and within the team. The theme also contains the subtheme “Suitability of video consultations.”

When communicating with patients, all teams reported on the use of phone and text messages. However, not all patients have consistent access to phones. The coordinator of Team 3 wished that the text messages would go directly into the EHR system, because those messages may contain important information. For communication with other partners, the FACT teams reported the use of standard solutions such as phone, video meetings, messages in the EHR systems, and standardized electronic messages. For communication within the teams, the teams reported the use of phone calls, text messages, video meetings, and messages in the EHR system. For distributed teams, video conference was used during the daily whiteboard meetings.

A subtheme in this category was suitability of video consultations. Teams 1, 3, and 5 used video conferencing to communicate with their patients. However, these teams emphasized that this is not suitable for all patients or all situations. One team leader said that suitability depends on how well he knows the patient: “It works well if I know the patient, but it is not suitable for a first meeting.”

Some teams were not using video conferencing. One reason for this is that they did not believe it would suit their patients. One team leader stated paranoia in patients as an issue. Patient access to a phone and internet to have a video consultation was another challenge. The team leader of Team 1 said that video conference had clear advantages over use of the phone, since it adds visual cues to the conversation. For instance, this allowed him to see if the patient was intoxicated. The use of video conference was mostly planned, but there were also examples of use in acute situations. One team reported the used of email when communicating with a patient, at the patient’s request.

#### Documentation

The Documentation theme describes various documentation issues, not necessarily directly related to the existing technological solutions. The team leader of Team 1 reported on different cultures for documentation for specialist and primary care, with team members employed in specialist care more used to extensive documentation. For FACT youth teams specifically, one issue that was brought up was the dilemma of what information should be included in the EHR documentation about family members. Family issues may impact youth and may thus be relevant information for documentation in the EHR. However, the team’s patient is the youth and not the family members. In addition, from these patients are 16 years old they can access their own patient journals, allowing them to read what has been documented. One team member in Team 6 stated:

This is a very long discussion, that we discussed for many, many, many hours in mental health for children and youth. It is not irrelevant if Mom has her issues, when the children are in the condition they are in. It is hard to know how to balance it. What can be written in the child’s journal, that the child at some point will have access to.

Most of the issues with documentation fell within the subtheme “Documentation while traveling.” FACT teams are highly mobile, which leads to challenges with documentation. Many teams reported that documentation is often postponed to the next day when they are traveling. Some teams had laptops that enabled them to document while traveling, and some reported completing the documentation when at various locations or at home. No teams documented while they were meeting with the patient. The team leader of Team 2 wished to be able to write in the EHR while with the patient, as this would be a good example of user involvement. Conversely, one team member in Team 7 was worried that documentation while with the patient would take the focus away from the patient.

#### Organization

Various organizational issues were brought up by the FACT teams. General issues included having access to ICT systems and being assigned the correct roles in the systems. The coordinator for Team 5 stated that she was surprised that there were not clearer guidelines for how FACT teams should operate.

A subtheme in this category was “Systems not adapted to FACT teams.” Several team leaders and coordinators stated that various systems are not tailored to FACT. This applied to both ICT solutions and organizational issues. One example was that the templates and roles that are defined in the specialist EHR are not suited for FACT. Some teams also expressed that the primary care EHR is more suited for somatic health and home care services. A team leader said that an issue with treatment plans is that they are focused on treatment, whereas FACT has a recovery focus. In addition, the issues with EHR access described below are related to this subtheme. Issues with standardized patient pathways are also examples of systems not adapted to FACT. The leader of Team 4 said that he supports standardized patient pathways, as they ensure that the patients receive the treatment they are entitled to. However, he thought that the patient pathways are poorly suited to the patient group. The main issue is that it can be difficult to reach the patients, and this is hard to show in the documentation of the pathways. The leader of Team 2 said that the patient pathways lead to evaluations of the patients that are not necessary. Some teams also want the patient pathways to be reflected in the electronic whiteboard, such as by showing deadlines related to the patient pathways. One team coordinator stated that one of the problems is that FACT is so different from other aspects of health care. FACT teams are team-based and use assertive outreach, which sets them apart from most of health care. They also focus on recovery, making it hard to document in systems that are focused on treatment, are part of mental health care, and often use ICT systems that are more suited for somatic health care.

#### COVID-19

The theme COVID-19 describes the various impacts the COVID-19 pandemic had on the teams at the time the interviews and observations were conducted. This theme was divided into the subthemes “Maintaining team capacity” and “Maintaining contact with patients.”

The subtheme of maintaining team capacity focuses on the organizational changes the teams made to protect the team members from COVID-19 and to maintain the teams in operation. One measure some FACT teams adopted was to divide the team into two groups, with one group working from home while the other group was at the office. This was done to prevent the risk of the whole team having to be quarantined at the same time due to being exposed to an infected person. The teams also used standard precautions such as face masks and maintaining physical distance from each other. One team leader stated that it was challenging because they had team members that were in the risk group for more serious COVID-19 complications themselves.

The subtheme of maintaining contact with patients describes the adaptations the teams had to make to follow up the patients while COVID-19 restrictions were in place. One team leader reported that it took a long time before they obtained acceptance for the need of following up their seriously ill patients in person. One team leader said that they met patients outdoors taking a walk to reduce the risk of infection. Several teams reported that they increased the use of the phone to reach their patients. The leader of Team 3 said that their patients made themselves more available by borrowing phones from friends or relatives, and that this made the patients almost more available than usual. Team 5 was already using video consultations before the pandemic hit. Team 1 was planning to start using video, and this process was accelerated so that the team started using video consultations during the pandemic. The teams described different reactions from the patients to the pandemic. One team talked about patients who did not relate to COVID-19, while another team described their patients as “scared” regarding the pandemic.

#### Technologies

##### General Components of the Theme

This category describes experiences with the various technological solutions the FACT teams use, including the subcategories EHRs, electronic whiteboards, calendars, and lack of integration. Some general issues that the teams described were obtaining access to computers; one team said it took 3 years before they got laptop computers. The teams also described various technical issues in getting the solutions to work. The FACT teams use questionnaires to map the status of their patients. Team 1 had a project where they wished to test the solution Checkware [25] for digital measures. Other teams were considering this solution as well.

##### Electronic Health Records

Most teams reported that they have employees from both primary and specialist care. All teams primarily documented in the specialist EHR system DIPS. To obtain access to DIPS, team members in primary care had so-called “simplified employment” or “0% positions” in specialist care. This means that they are considered to be employed in specialist care, granting them organizational access to the EHR data. In Team 4, all team members were employed in specialist care. The team leader of this team said he wished that they had access to the primary care EHR for a complete picture of the treatment the patient has received. In Team 2, all team members documented in DIPS. The team leader for this team said this led to their work not being seen by the municipality and was worried that this might lead to a cut in funding. In Team 3, the members were able to read information from the primary care EHR and found this to be useful, such as by obtaining information of any medication that was given to the patient from home care services. These perspectives show that access to EHR data from both primary and secondary care is important for the FACT teams and should be facilitated.

##### Electronic Whiteboard

The teams used various electronic whiteboard solutions, but all of them were based in Microsoft Excel. Several informants said that they wished the electronic whiteboards were integrated into the EHR or part of the EHR. The coordinator of Team 3 stated that they perform double documentation, both in the EHR and electronic whiteboard. Several informants said they had minor annoyances and issues and found the solutions hard to use. One suggestion to make the electronic whiteboard easier to use was color-coding of the information. Patients in Norway have the right to access their EHR information through the government health portal Helsenorge.no, but the leader of Team 4 said that the patients cannot access information on the electronic whiteboards in the same way.

Two of the FACT youth teams said they would like to be able to extract statistical information from the electronic whiteboards. One reason for this was to show the results they are obtaining to justify their funding. Team 7 said they wanted information about the number of patients, number of patients on compulsory treatment, number of patients with specific diagnoses, and number of patients who receive follow-up from child welfare services.

##### Calendar

The teams reported that they use different calendar systems, both in DIPS and Microsoft Outlook. The teams can have access to different versions of Microsoft Outlook, both from specialist care and from the municipalities. The DIPS calendar is used by many teams for reimbursement. The leader of Team 5 said that Skype appointments are automatically input into the Outlook calendar, but that these must be manually added to the DIPS calendar. She also said that the calendars are important because of the unpredictability of the work, with many changes that can happen during a day.

Some teams want to have a better team calendar to keep track of where the team members are. One reason for this is the added safety of knowing where the team members are when they are traveling to visit patients. Other team leaders stated that they already have a good overview of where their team members are and have routines regarding safety, so that they do not need a better calendar system for this purpose.

##### Lack of Integration

A common theme for the EHRs, electronic whiteboards, and calendars was the lack of integration between these solutions. There is also a lack of integration between the different EHR systems, making it hard to exchange information between the systems. A consequence of this is that team members wish to access the relevant EHR directly, leading to the challenges to access described above for the EHR subtheme.

There were also several team members that wished that the electronic whiteboards could exchange information with the EHR systems. One team leader described a need to jump between the EHR system and the electronic whiteboard that could be avoided with integration. Some EHR systems have built-in calendars, but the systems lack integration toward other calendar solutions.

## Discussion

### Principal Findings

In this study, we have explored the experiences and expectations of the FACT teams with the ICT tools that are relevant for their work. We found issues with communication, documentation, and organization. There were also some unique challenges with the COVID-19 pandemic and issues with the various technological solutions used by the teams, mainly related to integration and access to data.

One topic brought up in the analysis was the suitability of video consultations. Some teams used video consultations, but they did not think this was a suitable solution for all patients. Access to equipment and internet connection was also an issue for some patients. These results are similar to those of other studies on the use of video conferencing with patients of ACT and FACT teams during the COVID-19 pandemic [12,13], and are also consistent with findings on the use of video conferencing in multidisciplinary team–based treatment in somatic care [15]. This finding also matches a study on adaptations of the FACT model when implemented in rural areas in Norway, which showed that use of video meetings and video consultations were some of the adaptations made [7]. A study on therapists’ experiences of the use of video consultation in specialist mental health care during the COVID-19 pandemic highlighted that the therapists were hesitant to talk about the most sensitive themes and subjects. This was related to expectations or fear that the consultation would be interrupted by technical issues [26]. Use of video conference compared to phone calls has been documented as a better solution leading to fewer errors and better decision-making accuracy [27]. This has also been shown in our study, where one team leader reported that video conference had advantages over the use of phone calls, for instance by seeing if the patient was intoxicated. The successful use of video conference in some teams shows that it can be a useful tool for some patients, especially for teams with long travel distances.

Issues with documentation were mostly related to travel to meet the patients. Many eHealth solutions aim to reduce or eliminate the need for the patient or health care worker to travel [28]. However, due to the assertive outreach of FACT teams, traveling is an integral part of their work. Team workers often postpone documentation in the EHR, especially when they have long travel times. Some advantages and disadvantages of completing the documentation while with the patients were brought up in the interviews. On the one hand, letting the patient participate in documentation can be a good example of patient involvement. On the other hand, if done wrong, this can take the focus away from the patient during the meeting. The issues related to traveling also highlight that the ICT systems for FACT teams should be available on mobile devices such as smartphones and tablets. One issue that was unique to FACT youth teams was the dilemma regarding the type of health information that should be documented for family members in the youth EHR. While this information can be relevant for the youth, the health information for other individuals must be kept confidential.

Various organizational issues were brought up during the interviews. One common theme was that many ICT systems and ways of working are not adjusted to FACT teams. This is a consequence of the organization of most teams in both primary and secondary care, and the fact that the outreach model of FACT teams does not match the structure of many systems within health care.

The COVID-19 pandemic brought many challenges that forced the FACT teams to change the way they were working. Some of these changes were based on precautions that were common in other settings, such as the use of face masks and social distancing. A concern for some teams was that all team members would be quarantined at the same time due to being exposed to an infected person. The pandemic also changed the ways the teams were communicating with the patients, with increased use of phone and video consultations. For some teams, the implementation of video conferencing was accelerated because of the pandemic. These findings are similar to those of other studies on adaptations made by ACT and FACT teams during the COVID-19 pandemic [12,13]. These studies reported on various adaptations made during the pandemic, such as dividing the teams into two groups and increased use of telephone and video when communicating with patients.

The teams brought up various issues with the technologies they use. Access to EHR information was challenging for most teams, mainly because of the different EHR systems used in specialist and primary care. There were also challenges related to the electronic whiteboards. The main issue is the lack of integration between the electronic whiteboards and other systems such as EHRs. Integration could allow the sharing of data such as patient IDs, health care worker IDs, and diagnoses. Electronic whiteboards should also be able to extract statistical information that can be used for administrative purposes. FACT youth team members said they wished that the electronic whiteboards had a larger focus on family and network, even if the whiteboards in use have basic text fields for this. This was not brought up by the teams targeting adults, and thus reflects the increased focus on family and network in FACT youth teams. There was also a wish for electronic whiteboards to display information about deadlines related to standardized patient pathways.

There are several different calendar solutions available to FACT teams, but some teams still want one calendar system that gives an overview of the teams’ appointments. This is useful for administrative purposes and there is also safety in knowing where the team members are at any given time. However, the teams varied to the degree that they considered this to be a relevant issue. For example, this was not an issue focused on by the FACT youth teams.

The main challenge with respect to the ICT systems is a lack of integration between the various systems and access to relevant data. These issues are not unique to FACT teams, and were pointed out in the government strategy document One citizen–One Journal [29] in 2012. There is a need for EHR systems where FACT team members and other health care workers can easily access the relevant EHR data needed for their work. The electronic whiteboards should have integration toward these EHR systems.

### Limitations

One limitation of this study was that different methods were used in the data collection for teams targeting adults and youth. This was because of the availability of the teams and the added goals of the data collection for youth to serve as the basis of a requirement specification for an electronic whiteboard. At the same time, the focus on the teams’ ICT solutions was the same.

Another limitation is the choice of a theoretical, top-down data analysis approach. This limited our ability to perform an explorative analysis of all data, but allowed greater focus on the specific goals of our study.

### Conclusions

Despite the FACT model being successfully implemented in Norway, there are several issues regarding the ICT solutions they use, mainly related to access to data and integration. Further research is required to detail how improved ICT solutions should be designed. We are working on proposing an ideal solution for an electronic whiteboard, including a requirement specification and a description of how the electronic whiteboard should integrate toward EHR systems. While FACT teams targeting adults and youth differ in some ways, their needs for ICT solutions are largely similar. Some differences between the types of teams are a greater focus on family and network in teams targeting youth, and the challenges regarding information about family members in the youth EHRs. Regarding ICT solutions for FACT teams, these differences should be minor, and it should be possible to accommodate for the differences with configurations within the solutions.

## Abbreviations

ACT: Assertive Community Treatment
CSCW: computer-supported cooperative work
EHR: electronic health record
FACT: Flexible Assertive Community Treatment
ICT: information and communication technology

## Data Availability

The data sets generated during the current study are not publicly available due to the consent forms with our informants not allowing for it, but they are available for validation of our results from the corresponding author on reasonable request.
